# “Intention to receive COVID-19 vaccine among healthcare workers: a comparison between two surveys”

**DOI:** 10.1186/s12913-022-08379-3

**Published:** 2022-08-01

**Authors:** Alipasha Meysamie, Elham Ghasemi, Shadi Moshksar, Mehrdad Askarian

**Affiliations:** 1 Department of Community and Preventive Medicine, School of Medicine, Tehran University of Medical Sciences, Tehran, Iran; 2 Community-Based Participatory Research Center, Tehran University of Medical Sciences, Tehran, Iran; 3 Department of Community Medicine, School of Medicine, Shiraz University of Medical Sciences, Karimkhan-e Zand Avenue, Shiraz, 7134845794 Iran; 4 Health Behavior Science Research Center, Shiraz University of Medical Sciences, Shiraz, Iran

**Keywords:** COVID-19 vaccine, Intention, Health belief model, Healthcare workers

## Abstract

**Background:**

Considering the importance of intention to receive COVID-19 vaccine among healthcare workers and its role in maintaining their health and inhibiting the epidemic spread of Covid-19, the present study was done to identify the changes in intention to receive COVID-19 vaccine rate in two different time points and it’s determinants based on the dimensions of the health belief model among healthcare workers in Iran.

**Methods:**

Two cross-sectional surveys performed to investigate COVID-19 vaccination intent and associated factors based on the health belief model. The first conducted on 1244 participants from August 18 to 23, 2020, and the second on 1514 participants from February 5 to April 29, 2021, both using a questionnaire of intent to accept COVID-19 vaccination. The questionnaire distribution platform in both surveys was similarly, WhatsApp and Telegram social and working virtual groups of HCWs. Data were analyzed with SPSS-16 software for descriptive and analytical statistics.

**Results:**

In the first survey, 58.4% (95% CI: 0.55-0.61%) of healthcare workers intended to receive the COVID-19 vaccine, the rate dropped to 45.7% (95% CI: 0.43-0.48%) in the second survey (*P* < 0.001). The regression analysis indicated six factors that were significantly associated with higher intention to receive COVID-19 vaccine: being a female (OR = 1.84, 95% CI (1.11-3.03)), history of Covid-19 infection (OR = 1.54, 95% CI (1.09-2.18), perceptions of Covid-19 disease (OR = 1.13, 95% CI (1.01-1.28)), perceived benefits of COVID-19 vaccine (OR = 1.34, 95% CI (1.22-1.47)), prosocial norms for COVID-19 vaccination (OR = 1.25, 95% CI (1.21-1.29)), and COVID-19 vaccine safety/cost concerns (OR = 1.25, 95% CI (1.17-1.33)).

**Conclusions:**

Present study showed an undesirable rate of intention to receive COVID-19 vaccine among healthcare workers, especially decreasing over the time, emphasize the need of interventions to promote healthcare workers’ intention to receive the vaccine and reduce the spread of COVID-19 disease.

## Background

According to the statement of the director of the World Health Organization, the COVID-19 outbreak constitutes a Public Health Emergency of International Concern (PHEIC) on 30 January 2020 and a Pandemic on 11 March 2020 [1]. This pandemic has had a profound impact on the health of the worldwide community, economy, and social behavior. Despite all efforts since the beginning of the pandemic, definitive treatment for the disease has not yet been approved and vaccine development efforts are among the most effective priorities to save human lives [2, 3].

Vaccine provision for communities is very important to respond to COVID-19, but vaccine hesitancy and the reluctance to vaccination can play as an effective barrier, limiting efforts to control the pandemic. Responsibility of governments to the widespread distribution of the vaccine and its equitable access to prevent the spread of COVID-19 requires the necessary capacity of health systems and the use of appropriate methods to improve vaccine trust and acceptance to be effective [3].

Despite the evidence for the safety and effectiveness of vaccines, there is still hesitancy about their uptake in different communities. This can be problematic in building herd immunity [4, 5]. People are concerned about vaccines’ efficacy and side effects, and reluctance to get vaccinated significantly impacts the success of vaccination and pandemic control programs [6]. Given the importance of this issue, numerous studies worldwide have examined the willingness or intention of individuals to receive the COVID-19 vaccine. According to a systematic review, the willingness to get the COVID-19 vaccine has been estimated from 27.7% in the Congo to 91.3% in China [3]. A Rapid Systematic Review and Meta-Analysis conducted by Patwary et al. showed that the pooled-effect size of the COVID-19 vaccine acceptance rate was 58.5% (95% CI: 46.9, 69.7, I2 = 100%, 33 studies) and the pooled vaccine hesitancy rate was 38.2% (95% CI: 27.2–49.7, I2 = 100%, 32 studies) [5]. Another systematic review of the PubMed/Medline database also reported the rate of acceptance of the COVID-19 vaccine in adults in the general population, from 97% in Ecuador to 23% in Kuwait, and in the population of healthcare workers (HCWs) from 27.7% in Congo to 78.1% in Israel [7]. These studies discuss vaccination hesitancy a serious problem in the management and control of the COVID-19 pandemic.

HCWs are one of the high-risk groups for the disease, and several countries have prioritized them as of the first phase of their vaccination programs [8]. COVID-19 vaccination in Iran began in February 2021 with a limited number of Russian Sputnik-V vaccines, and HCWs and medical personnel were the first groups to receive the COVID-19 vaccine [9]. COVID-19 infection in this group has many negative consequences for the health systems. Disease and mortality of HCWs can lead to a serious crisis in the staffing of health care providers and inefficiency in epidemic management. On the other hand, given that these people are in frequent contact with colleagues, health service recipients and family members, they can quickly transmit the virus to the others [10, 11]. Vaccine acceptance among HCWs not only protects them against the disease but also helps to build public trust in vaccines, as they are a reliable source for promoting credible information about the importance of vaccines [12, 13]. Therefore, understanding factors related to the HCWs’ intention to accept or reject the COVID-19 vaccine is critical to successful vaccination planning and disease control.

As one of the most widely models of health behavior applied, the health belief model (HBM) has been used in various studies to express and predict preventive health behavior of the people. This model is based on the hypothesis that preventive behavior is based on the beliefs and includes 5 main constructs: Perceived susceptibility (belief about the possibility of acquiring a disease), Perceived severity (the person’s feelings about the severity of such a disease), Perceived benefits (One understands the usefulness of a particular health behavior), Perceived barriers (Assessing the barriers that can prevent a person from engaging in a health behavior), and Cues to action (cues that trigger a particular health behavior) [14]. HBM has been widely used in vaccine research to study vaccination behaviors and identify people’s understanding of the disease and vaccination [15].

The vaccine acceptance and hesitancy, as complex contexts, are affected by multiple factors such as time, place, social behavior of the society and specific vaccines [13, 16]. In developing countries, such as Iran, there are several cultural, political, social, and economic challenges affecting the Covid-19 vaccination. One of the challenges that has affected the vaccination of medical personnel is the lack of trust that caused less confidence about the vaccines’ effectiveness and side effects [9]. In terms of predicting vaccine intent, identifying important HBM items which affect the intent, can be an important step in increasing vaccine coverage [17], and according to the researches in this field, this model has been approved as a useful model in identifying the intention to perform COVID-19 vaccine [4, 15, 18–20]. Therefore, the present study aimed to investigate the intention of Iranian HCWs to receive the COVID-19 vaccine based on HBM constructs.

## Methods

Two cross-sectional surveys performed to investigate COVID-19 vaccination intent and associated factors based on health belief model. A questionnaire were distributed widely from the 18th to 23rd of August 2020 for the first round, and about 6 months later from 5th of February to 29th of April 2021 for the second round. The distribution platform in both surveys was similarly, WhatsApp and Telegram social and working virtual groups of HCWs. Sampling technique was purposeful convenient sampling and asked those who received the message to distribute the questionnaire to other colleagues with a snowball sampling technique. For the second survey the questionnaires were sent to the same groups and they were asked to share with the same colleagues as distributed previously. Sample size calculation performed according to estimating the willingness to vaccination about 50% with 3% acceptable difference and 5% type one error according to the online sample size calculator [21] and was 1064. By considering 10% missing data, calculated about 1201 for each round.

The questionnaire consisted of three main sections. The first one included demographic information: age, gender, pregnancy status, current location, marital status, the highest level of education, history of chronic disease related to the severity of the COVID-19 (including diabetes mellitus, hypertension, lung diseases, renal diseases, cardiovascular diseases, and corticosteroids consumption), history of COVID-19 infection, history of COVID-19 disease among friends, colleagues or family members, and history of flu vaccination.

The second section, HBM domains, consisted of 26 items which were validated previously through factor analysis [22]. These 26 items were categorized into six factors encompassing: perceptions of COVID-19 disease (three items), perceived benefits of vaccination (four items), COVID-19 vaccine safety/cost concerns (four items), preferences for COVID-19 vaccine alternatives (four items), prosocial norms for COVID-19 vaccination (eight items), and COVID-19 risk-reduction habits (three items). All items were answered in a 5-point Likert scale from ‘Strongly disagree’ (score: 1) to ‘strongly agree’ (score: 5). The results of reliability investigation of COVID-19 related health belief model domains are presented in Table 1.

**Table 1:** Internal consistency (Cronbach’s alpha) and Intraclass reliability of COVID-19 related health belief model domains (*n* = 2756)

COVID-19 related health belief model scale		Number of items	Cronbach’s Alpha Coefficients	Intraclass Correlation (ICC)	Intraclass Correlation (ICC) CI 95%	*P* value
Domains	Perceptions of COVID-19 disease	3	0.613	0.576	0.548-0.603	< 0.001
	Perceived benefits COVID-19 vaccine	4	0.972	0.971	0.969-0.973	< 0.001
	COVID-19 vaccine safety/cost concerns	4	0.741	0.739	.723-0.755	< 0.001
	Preference for COVID-19 vaccine alternatives	4	0.665	0.626	0.602-0.648	< 0.001
	Prosocial norms for the COVID-19 vaccination	8	0.924	0.922	0.918-0.927	< 0.001
	COVID-19 risk-reduction habits	3	0.816	0.816	0.803-0.827	< 0.001

The third section was the primary outcome measure of intention to receive COVID-19 vaccination. This section had one major item, “I will get the COVID-19 vaccine as soon as it is accessible”. The answer was a 5-point Likert scale ranging from one (strongly disagree) to five (strongly agree). For the analysis, the scale has been transformed from 5-point scale to the binomial values of ‘Yes’ consisted of ‘Strongly agree’ and ‘Agree’ answers and the others as ‘No’. In addition, there was another item in this section that asked for the kind of vaccine they prefer (Foreign vaccine, domestic vaccine, or it makes no differences).

### Statistical analysis

Data analysis was performed by using SPSS software version 16 [23]. A *P*-value < 0.05 was considered statistically significant. Descriptive statistics were reported using frequency and percentages for categorical data, and mean and standard deviation for numeric data. Independent sample t-test and chi-square test were used for quantitative and qualitative variables comparison between groups intended to vaccination and non-intended to vaccination, respectively. The logistic regression analysis was performed to determine the relationship between independent variables (HBM domains, and personal characteristics) and outcome variable (the intention to receive the COVID-19 vaccine). Reliability analysis performed by measurement of chronbachs alpha and intraclass correlation coefficient (ICC) for each domains of the HBM questionnaire.

### Ethical considerations

This study was approved by the ethics committee of Shiraz University of Medical Sciences with the ethical codes IR.SUMS.MED.REC.1399.276 and IR.SUMS.MED.REC.1399.549 for first and second rounds respectively. The informed consent was obtained from all participants in two surveys according to the first informed question, if any person disagreed, automatically redirected to a page as exit from the study. In the consent questions ethical considerations were observed, including providing the necessary explanations about the objectives and the method of the study, confidentiality of the information, the right to withdraw the study at any time. There was no reward for completion of the questionnaire.

## Results

### Sample characteristics in two surveys

The sample included HCWs, 1244 in the first survey and 1514 in the second round. The mean age of the sample in the first and second surveys was 41.4 ± 11.9 and 40.1 ± 10.3, respectively (*P* = 0.027). The highest frequency of samples in both surveys belonged to Fars and Tehran provinces. In the second study, compared with the first study, participants had a higher history of COVID-19 infection as well as in their friends/colleagues/relatives, and a higher history of influenza vaccine injection; moreover the percentage of unmarried and postgraduate people was higher (*P* < 0.001). Comparison of characteristics between samples are presented in Table 2.

**Table 2:** Comparison of the characteristics of participants in the two surveys

Variable		First survey (August 2020) *n* = 1244	Second survey (February 2021) *n* = 1512	*P* value*
Gender	Male	423 (34%)	515 (34.1%)	0.975
	Female	821 (66%)	997 (65.9%)	
Pregnancy	Yes	25 (3%)	21 (1.9%)	0.087
	No	796 (97%)	1111 (98.1%)	
Marital Status	Single	937 (75.3%)	1176 (77.8%)	< 0.001
	Married	307 (24.7%)	317 (21%)	
	Other Status	0 (0%)	19 (1.3%)	
Education	Lower than high school diploma	6 (0.5%)	3 (0.2%)	< 0.001
	High school diploma	61 (4.9%)	39 (2.6%)	
	Associate degree	42 (3.4%)	38 (2.5%)	
	Bachelor	349 (28.1%)	732 (48.4%)	
	MSc	166 (13.3%)	116 (7.7%)	
	PhD and above	620 (49.8%)	580 (38.4%)	
History of chronic disease / Corticosteroid use	Yes	190 (15.3%)	236 (15.6%)	0.809
	No	1054 (84.7%)	1276 (84.4%)	
History of COVID-19 infection	Yes	204 (16.4%)	548 (36.2%)	< 0.001
	No	1040 (83.6%)	964 (63.8%)	
History of COVID-19 infection in friends, colleagues, and relatives	Yes	953 (76.6%)	1471 (97.3%)	< 0.001
	No	291 (23.4%)	41 (2.7%)	
History of receiving the flu vaccine	Yes	657 (52.8%)	971 (64.2%)	< 0.001
	No	587 (47.2%)	541 (35.8%)	

As shown in Table 3, in terms of the “perceptions of COVID-19 disease”, “COVID-19 vaccine safety/cost concerns”, and “preference for COVID-19 vaccine alternatives” dimensions, the second survey sample had a higher scores (*P* < 0.001); However, the scores obtained in the dimensions of “perceived benefits of COVID-19 vaccination” (*P* = 0.001) and “prosocial norms for COVID-19 vaccination” (*P* < 0.001) were significantly higher in the first survey than in the second survey. Participants of the two surveys were not statistically significant different in “COVID-19 risk-reduction habits” domain (*P* = 0.578).

**Table 3:** Comparison of the two groups of the participants in terms of COVID-19 related health belief domains

Domain	Item	Survey	Strongly disagree	Disagree	No idea	Agree	Strongly agree	*P*-value
Perceptions of COVID-19 disease	1. COVID-19 is an important and serious disease.	1st (*n* = 1244)	15 (1.2%)	9 (0.7%)	19 (1.5%)	329 (26.4%)	872 (70.1%)	< 0.001^ε^
		2nd (n = 1512)	1 (0.1%)	2 (0.1%)	13 (0.9%)	236 (15.6%)	1259 (83.3%)	
	2. I am very susceptible to this disease.	1st (*n* = 1244)	51 (4.1%)	213 (17.1%)	326 (26.2%)	470 (37.8%)	184 (14.8%)	< 0.001^ε^
		2nd (*n* = 1512)	21 (1.4%)	122 (8.1%)	247 (16.3%)	646 (42.7%)	476 (31.5%)	
	3. If I get the disease, people who directly contact me will be more likely to get it.	1st (*n* = 1244)	5 (0.4%)	31 (2.5%)	57 (4.6%)	549 (44.1%)	602 (48.4%)	< 0.001^ε^
		2nd (*n* = 1512)	2 (0.1%)	16 (1.1%)	35 (2.3%)	514 (34.0%)	945 (62.5%)	
	Domain score (Mean ± SD)	1st (*n* = 1244)	12.4 ± 1.8					< 0.001^ɸ^
		2nd (*n* = 1512)	13.3 ± 1.6					
Perceived benefits of COVID-19 vaccine	4. I will definitely get vaccinated if I know that getting the COVID-19 vaccine will protect me against this disease.	1st (*n* = 1244)	17 (1.4%)	22 (1.8%)	47 (3.8%)	301 (24.2%)	857 (68.9%)	0.012^ε^
		2nd (*n* = 1512)	23 (1.5%)	36 (2.4%)	87 (5.8%)	413 (27.3%)	953 (63.0%)	
	5. If I know that getting the COVID-19 vaccine protects my family and friends, I will definitely get the vaccine.	1st (*n* = 1244)	15 (1.2%)	17 (1.4%)	35 (2.8%)	283 (22.7%)	894 (71.9%)	0.003^ε^
		2nd (*n* = 1512)	15 (1.0%)	36 (2.4%)	71 (4.7%)	389 (25.7%)	1001 (66.2%)	
	6. If I know that getting a COVID-19 vaccine protects other people in the community, I will definitely get vaccinated.	1st (*n* = 1244)	13 (1.0%)	19 (1.5%)	41 (3.3%)	300 (24.1%)	871 (70.0%)	0.02^ε^
		2nd (*n* = 1512)	17 (1.1%)	36 (2.4%)	78 (5.2%)	397 (26.3%)	984 (65.1%)	
	7. If I know that getting the Corona vaccine will bring society back to normal, I will definitely get the vaccine.	1st (*n* = 1244)	13 (1.0%)	14 (1.1%)	30 (2.4%)	261 (21.0%)	926 (74.4%)	< 0.001^ε^
		2nd (*n* = 1512)	13 (0.9%)	29 (1.9%)	72 (4.8%)	371 (24.5%)	1027 (67.9%)	
	Dimension score (Mean ± SD)	1st (*n* = 1244)	18.5 ± 2.7					0.001^ɸ^
		2nd (*n* = 1512)	18.1 ± 3.0					
COVID-19 vaccine safety/cost concerns	8. I am concerned about the side effects of the COVID-19 vaccine.	1st (*n* = 1244)	30 (2.4%)	123 (9.9%)	229 (18.4%)	511 (41.1%)	351 (28.2%)	< 0.001^ε^
		2nd (*n* = 1512)	10 (0.7%)	56 (3.7%)	114 (7.5%)	433 (28.6%)	899 (59.5%)	
	9. I think the COVID-19 vaccine might be dangerous for me.	1st (*n* = 1244)	85 (6.8%)	313 (25.2%)	408 (32.8%)	324 (26.0%)	114 (9.2%)	< 0.001^ε^
		2nd (*n* = 1512)	24 (1.6%)	166 (11.0%)	272 (18.0%)	522 (34.5%)	528 (34.9%)	
	10. I’m worried about getting COVID-19 from this vaccine.	1st (*n* = 1244)	309 (24.8%)	365 (29.3%)	302 (24.3%)	217 (17.4%)	51 (4.1%)	< 0.001^ε^
		2nd (*n* = 1512)	109 (7.2%)	327 (21.6%)	316 (20.9%)	418 (27.6%)	342 (22.6%)	
	11. This vaccine will cost me a lot of money.	1st (*n* = 1244)	211 (17.0%)	320 (25.7%)	392 (31.5%)	252 (20.3%)	69 (5.5%)	< 0.001^ε^
		2nd (*n* = 1512)	224 (14.8%)	510 (33.7%)	479 (31.7%)	195 (12.9%)	104 (6.9%)	
	Dimension score (Mean ± SD)	1st (*n* = 1244)	12.1 ± 3.2					< 0.001^ɸ^
		2nd (*n* = 1512)	14.3 ± 3.2					
Preference for COVID-19 vaccine alternatives	12. I am afraid of injections.	1st (*n* = 1244)	681 (54.7%)	395 (31.8%)	67 (5.4%)	78 (6.3%)	23 (1.8%)	< 0.001^ε^
		2nd (*n* = 1512)	614 (40.6%)	580 (38.4%)	141 (9.3%)	113 (7.5%)	64 (4.2%)	
	13. I believe in natural remedies and traditional medicine to treat COVID-19.	1st (*n* = 1244)	580 (46.6%)	297 (23.9%)	224 (18.0%)	107 (8.6%)	36 (2.9%)	< 0.001^ε^
		2nd (*n* = 1512)	559 (37.0%)	407 (26.9%)	326 (21.6%)	157 (10.4%)	63 (4.2%)	
	14. I do not get the COVID-19 vaccine because of my religious beliefs.	1st (*n* = 1244)	1009 (81.1%)	210 (16.9%)	18 (1.4%)	2 (0.2%)	5 (0.4%)	< 0.001^ε^
		2nd (*n* = 1512)	946 (62.6%)	459 (30.4%)	88 (5.8%)	12 (0.8%)	7 (0.5%)	
	15. I do not get this vaccine because I am not part of the high-risk group.	1st (*n* = 1244)	763 (61.3%)	385 (30.9%)	55 (4.4%)	32 (2.6%)	9 (0.7%)	< 0.001^ε^
		2nd (*n* = 1512)	641 (42.4%)	608 (40.2%)	185 (12.2%)	51 (3.4%)	27 (1.8%)	
	Dimension score (Mean ± SD)	1st (*n* = 1244)	6.4 ± 2.3					< 0.001^ɸ^
		2nd (*n* = 1512)	7.4 ± 2.7					
Prosocial norms for the COVID-19 vaccination	16. I am getting this vaccine because I have heard about its benefits from national media programs.	1st (*n* = 1244)	112 (9.0%)	192 (15.4%)	362 (29.1%)	339 (27.3%)	239 (19.2%)	< 0.001^ε^
		2nd (*n* = 1512)	302 (20.0%)	347 (22.9%)	446 (29.5%)	300 (19.8%)	117 (7.7%)	
	17. I am getting this vaccine because I have read about its benefits on the internet and cyberspace.	1st (*n* = 1244)	78 (6.3%)	185 (14.9%)	341 (27.4%)	406 (32.6%)	234 (18.8%)	< 0.001^ε^
		2nd (*n* = 1512)	190 (12.6%)	300 (19.8%)	435 (28.8%)	404 (26.7%)	183 (12.1%)	
	18. I am getting this vaccine because I have heard about its benefits from a doctor I trust.	1st (*n* = 1244)	60 (4.8%)	163 (13.1%)	365 (29.3%)	405 (32.6%)	251 (20.2%)	< 0.001^ε^
		2nd (*n* = 1512)	162 (10.7%)	296 (19.6%)	488 (32.3%)	385 (25.5%)	181 (12.0%)	
	19. Most people who care about me think I should get vaccinated.	1st (*n* = 1244)	43 (3.5%)	127 (10.2%)	333 (26.8%)	495 (39.8%)	246 (19.8%)	< 0.001^ε^
		2nd (*n* = 1512)	97 (6.4%)	313 (20.7%)	443 (29.3%)	475 (31.4%)	184 (12.2%)	
	20. My trusted doctor thinks I should get vaccinated.	1st (*n* = 1244)	35 (2.8%)	111 (8.9%)	405 (32.6%)	453 (36.4%)	240 (19.3%)	< 0.001^ε^
		2nd (*n* = 1512)	94 (6.2%)	243 (16.1%)	519 (34.3%)	464 (30.7%)	192 (12.7%)	
	21. My family members think I should get vaccinated.	1st (*n* = 1244)	35 (2.8%)	102 (8.2%)	290 (23.3%)	512 (41.2%)	305 (24.5%)	< 0.001^ε^
		2nd (*n* = 1512)	116 (7.7%)	258 (17.1%)	373 (24.7%)	503 (33.3%)	262 (17.3%)	
	22. My friends think I should get vaccinated.	1st (*n* = 1244)	39 (3.1%)	109 (8.8%)	312 (25.1%)	494 (39.7%)	290 (23.3%)	< 0.001^ε^
		2nd (*n* = 1512)	108 (7.1%)	274 (18.1%)	438 (29.0%)	462 (30.6%)	230 (15.2%)	
	23. I know that other people my age or my classmates get the vaccine.	1st (*n* = 1244)	21 (1.7%)	96 (7.7%)	359 (28.9%)	533 (42.8%)	235 (18.9%)	< 0.001^ε^
		2nd (*n* = 1512)	70 (4.6%)	274 (18.1%)	528 (34.9%)	472 (31.2%)	168 (11.1%)	
	Dimension score (Mean ± SD)	1st (*n* = 1244)	28.5 ± 6.7					< 0.001^ɸ^
		2nd (*n* = 1512)	25.3 ± 7.2					
COVID-19 risk-reduction habits	24. I follow health protocols based on the use of masks.	1st (*n* = 1244)	10 (0.8%)	2 (0.2%)	7 (0.6%)	203 (16.3%)	1022 (82.2%)	0.035^ε^
		2nd (*n* = 1512)	1 (0.1%)	5 (0.3%)	9 (0.6%)	258 (17.1%)	1239 (81.9%)	
	25. I follow health protocols based on physical distancing.	1st (*n* = 1244)	4 (0.3%)	2 (0.2%)	14 (1.1%)	343 (27.6%)	881 (70.8%)	0.402^ε^
		2nd (*n* = 1512)	3 (0.2%)	4 (0.3%)	22 (1.5%)	374 (24.7%)	1109 (73.3%)	
	26. I follow hygienic protocols for frequent hand washing and disinfection of surfaces.	1st (*n* = 1244)	4 (0.3%)	4 (0.3%)	13 (1.0%)	267 (21.5%)	956 (76.8%)	0.931^ε^
		2nd (*n* = 1512)	4 (0.3%)	7 (0.5%)	16 (1.1%)	340 (22.5%)	1145 (75.7%)	
	Dimension score (Mean ± SD)	1st (*n* = 1244)	14.2 ± 1.4					0.58^ɸ^
		2nd (*n* = 1512)	14.2 ± 1.3					

ε: Chi-squared test

ɸ: Independent sample t-test

In terms of vaccine type preferences, the results showed that the proportion of HCWs who preferred the foreign vaccine was higher in the first survey (Table 4). Regarding the primary outcome of the study (intention to receive the COVID-19 vaccine), 58.4% (95% CI:55.3-61.2%) of HCWs intended to receive the vaccine in the first survey. Still, this rate reached 45.7% (95% CI: 43.4-48.3%) in the second survey, and this difference was statistically significant (*P* < 0.001) (Table 5).

**Table 4:** Comparison of COVID-19 vaccine type preferences in two surveys

Item	First survey *n* = 1244			Second survey *n* = 1512			*P* value
	Foreign vaccine	Domestic vaccine	It does not matter	Foreign vaccine	Domestic vaccine	It does not matter	
COVID-19 Vaccine preferences	761	208	275	696	294	522	< 0.001
	(61.2%)	(16.7%)	(22.1%)	(46.0%)	(19.4%)	(34.5%)

**Table 5:** Comparison of intention to receive COVID-19 vaccine in two surveys

Item	First survey *n* = 1244		Second survey *n* = 1512		*P* value
	Disagree	Agree	Disagree	Agree	
I will be vaccinated as soon as the COVID-19 vaccine is available.	517	727	821	691	< 0.001
	(41.6%)	(58.4%)	(54.4%)	(45.7%)	

The results of comparing different dimensions of the HBM in participants of the two surveys by T test analysis, indicated that the scores of “COVID-19 vaccine safety/cost concerns” and “preference for COVID-19 vaccine alternatives” dimensions in the group that did not intend to receive the vaccine were significantly higher than the group that intended to receive the vaccine. While the scores of other dimensions of the HBM were higher in the group that intended to receive the vaccine in comparison with the group that did not intend to receive the vaccine (*P* < 0.001) (Table 6). Comparison of HBM dimensions in two groups with and without the intention to receive the COVID-19 vaccine separately in two surveys also showed similar results.

**Table 6:** Intention to receive COVID-19 vaccine by domains of HBM (*n* = 2756)

Domains of HBM	Intention to receive COVID-19 vaccine		*P*-value ^ɸ^
	NO	YES	
Perceptions of COVID-19 disease	12.6 ± 1.8	13.2 ± 1.6	< 0.001
Perceived benefits of COVID-19 vaccine	17.5 ± 3.6	19.4 ± 1.4	< 0.001
COVID-19 vaccine safety/cost concerns	14.9 ± 2.7	11.8 ± 3.3	< 0.001
Preference for COVID-19 vaccine alternatives	7.6 ± 2.7	6.3 ± 2.3	< 0.001
Prosocial norms for the COVID-19 vaccination	22.6 ± 6.3	30.8 ± 5.5	< 0.001
COVID-19 risk-reduction habits	14.1 ± 1.4	14.4 ± 1.2	< 0.001

ɸ: Independent sample t-test

Table 7 presents the logistic regression of the factors associated with intention to receive COVID-19 vaccine. The results showed that being a female (OR = 1.84, 95% CI (1.11-3.03)), history of Covid-19 infection (OR = 1.54, 95% CI (1.09-2.18), perceptions of Covid-19 disease (OR = 1.13, 95% CI (1.01-1.28)), perceived benefits of COVID-19 vaccine (OR = 1.34, 95% CI (1.22-1.47)), prosocial norms for COVID-19 vaccination (OR = 1.25, 95% CI (1.21-1.29)), and COVID-19 vaccine safety/cost concerns (OR = 1.25, 95% CI (1.17-1.33)) were significantly associated with higher intention to receive COVID-19 vaccine.

**Table 7:** Logistic regression model for the factors associated with HCW’s intention to receive COVID-19 vaccine

Variable	B	S.E	P value	Exp (B)	95% CI for Exp (B)	
Gender	0.608	0.255	0.017	1.84	1.11	3.03
History of Covid-19 infection	0.431	0.178	0.015	1.54	1.09	2.18
Perceptions of Covid-19 disease	0.126	0.062	0.042	1.13	1.01	1.28
Perceived benefits of COVID-19 vaccine	0.292	0.046	< 0.001	1.34	1.22	1.47
Prosocial norms for COVID-19 vaccination	0.222	0.017	< 0.001	1.25	1.21	1.29
COVID-19 vaccine safety/cost concerns	0.221	0.032	< 0.001	1.25	1.17	1.33

## Discussion

According to this study, not only the proportion of HCWs intended to be vaccinated was low in both surveys (58.4 and 45.7%, respectively), but also the rate has been decreased in the 2nd survey subsequently. Because HCWs are at higher risk for COVID-19 infection and are responsible for prescribing and recommending vaccines to patients and the general population, it is crucial to identify their willingness for vaccination and other preventive measures, especially when incorrect information about the vaccine has been spread in the population [15]. Our findings are in line with the results of previous studies; for example, a systematic review and meta-analysis estimated the ratio of intention to receive the COVID-19 vaccine in HCWs at 55.9% (95% CI: 43.6-67.9%) and in the range of 27.7 to 81.5% [24]. Also, according to another systematic review, the acceptance rate of the COVID-19 vaccine in the population of HCWs has been reported from 27.7% in the Congo to 78.1% in Israel [7]. The wide range of intent or willingness to receive the COVID-19 vaccine in HCWs in different countries may be due to the influence of contextual factors on this issue. Hesitancy about vaccines has been raised as a matter of time and context [25]. However, what previous studies have in common is the need to address this issue and its importance in maintaining the health of HCWs and the whole population, thereby increasing the success of vaccination programs and preventing the spread of pandemics.

The findings indicated that respondents to the second survey had a higher perception of the risk of COVID-19, COVID-19 vaccine safety/cost concerns and preference for COVID-19 vaccine alternatives, as well as lower scores on perceived benefits COVID-19 vaccine and effect of social factors, including family, friends, media and physicians on the intention to receive the COVID-19 vaccine compared to the first survey. Considering that the second survey was conducted after the fourth peak of COVID-19 in Iran, therefore, it seems that due to the experience of increasing the number of cases, hospitalizations and death, HCWs have considered this disease more important and severe and also themselves susceptible to contracting and transmitting the disease or in other words, they had a higher understanding of the risk of COVID-19 disease. However, given that over time, there was still no certainty about benefits of the COVID-19 vaccine and its side effects, other ways to prevent Covid-19 may have been preferred to the vaccination. Low HCW’s intention to receive the COVID-19 vaccine over time and also high scores of the two dimensions of “COVID-19 vaccine safety/cost concerns” and “preference for COVID-19 vaccine alternatives” in the second survey compared to the first survey demonstrate the need for education and provision of valid and scientific information on COVID-19 disease, the effects of the vaccine, and its possible side effects among HCWs. As in a previous study, HCWs’ hesitancy about receiving the COVID-19 vaccine were reported due to incorrect information about the severe side effects of the COVID-19 vaccine [10]. An investigation of COVID-19 vaccine acceptance across nine Low- and Middle-Income Countries showed that the COVID-19 vaccine acceptance was positively associated with COVID-19 knowledge [26]. In addition, other researchers, based on the results of their systematic review and meta-analysis on the intention to receive the Covid-19 vaccine in HCWs, have emphasized the need for education efforts urgently to improve knowledge, attitude and practice to promote vaccination [27]. Previous studies have also confirmed the effectiveness of educational interventions on vaccine hesitancy [28] and attitudes toward COVID-19 vaccination acceptability [29].

In terms of HBM domains, the results of logistic regression showed that “perceptions of Covid-19 disease”, “perceived benefits of COVID-19 vaccine”, “prosocial norms for COVID-19 vaccination”, and “COVID-19 vaccine safety/cost concerns” were significantly associated with higher intention to receive COVID-19 vaccine. The HCWs who obtained higher scores on the perceptions of the COVID-19 disease, the benefits of the Covid-19 vaccine, the effects of prosocial norms on receiving the vaccine, and concerns about the side effects or costs of the vaccine were more likely to intend to receive the vaccine. Similar to the findings of the present study, the results of a systematic review also reported numerous evidences confirming the association between willingness to receive the COVID-19 vaccine and several factors such as perceived risk of COVID-19, perceived benefit of vaccine, perceived vaccine barriers, use of social media for COVID-19 vaccine-related information, recommended for vaccination, perceived effectiveness of a COVID-19 vaccine, and COVID-19 vaccine safety concerns [3]. Previous studies have also linked the dimensions of the HBM to the willingness or intention to receive COVID-19 vaccine; for example, the association of the willingness to receive COVID-19 vaccine with HBM domains (including Perceived benefits, Perceived barriers, cue to action as well as subjective norms) has been found among the HCWs population in Iraq [4]. Moreover, the positive association of COVID-19 vaccination intent with HBM factors including perceived severity, perceived benefits, self-efficacy, cues to action (via acquaintances and social media) in the HCWs population in China has been reported [20]. Paying attention to vaccine barriers and enablers, and intervening in this regard play an important role in improving the success of vaccination among HCWs [30]. In particular, addressing concerns about the side effects and effectiveness of the vaccine should be considered to increase the willingness to receive the vaccine [3, 31]. According to the results of a study on the willingness to accept the COVID-19 vaccine among physicians and nurses in France, Belgium, and Canada, 48.6% of participants had a high acceptance of the vaccine. In comparison, 23% had a moderate acceptance level, and 28.4% were hesitant or reluctant about the vaccine. In this group, concern about vaccine safety was identified as the most essential factor associated with hesitation or reluctance to receive the COVID-19 vaccine in HCWs [32].

According to the evidence, women account for 70% of healthcare workers and their role is still fundamental in the management of the COVID-19 pandemic [33, 34]. Therefore, our finding, which showed that 66% of the participants were female, is expected. In addition, the review of the literature related to the willingness or intention of HCWs to receive the COVID-19 vaccine showed similar findings; So that studies conducted in Iran [35, 36] and other countries [4, 13, 15, 20] reported that the majority of healthcare workers participating in these studies were female. Our results showed that females were 1.8 times more likely to intend to receive COVID-19 vaccine compared to males. This could be explained by a higher risk of exposure and infection and increased caregiving responsibilities among women during COVID-19 [37], and their more likelihood to seek out preventive health care. Our finding was consistent with a previous study that showed that females were more likely to agree with the statement that “getting myself vaccinated for COVID-19 would be a good way to protect myself against infection” than males (aOR = 1.4 (95% CI: 1.1–1.8); *P* = 0.03) [38].

Besides, history of COVID-19 infection was associated to intend to receive COVID-19 vaccine. This may be due to the fear of getting the disease again and thus more intention to vaccine uptake. Our finding was in line with the findings of a study conducted among adults in Nigeria, which showed that individuals without prior diagnosis of COVID-19 had less perception to receive COVID-19 vaccine (adjusted OR = 0.59 (95% CI: 0.38–0.92), *P* = 0.021) than those that were previously COVID-19 positive [39].

### Implication of study findings

Due to more scientific information HCWs have about the preparation and production of vaccines, such as the side effects and potential risks of vaccines, they are naturally more concerned than other groups of people, and for this reason, they may not be sure about the use of vaccines. Therefore, for the country’s HCWs, familiarization courses regarding COVID-19 vaccination, such as online educational workshops along with question and answer sessions, seems to be logical in the current situation, because of the other members of the society decision to receive or not to receive the vaccine, can be influenced by the HCWs.

### Strengths and weaknesses of the study

To the best of our knowledge, this study identified the intention to receive the COVID-19 vaccine among HCWs in two different period of time for the first time in Iran. Therefore, it can provide important information to take the necessary measures by decision-makers in this area. However, the present study has some limitations. The researchers applied convenient sampling method so the representativeness of the samples in both surveys may be limited. In addition, web-based and cyberspace navigation can have likelihood of participant bias. Considering the time of the surveys was related to before the start of the vaccination program in the country, and since the behavioral intent can change over time, similar studies in the future are suggested to provide the information needed to better understand the process of vaccination intent in HCWs.

## Conclusion

The present study results showed an undesirable level of intention to receive the COVID-19 vaccine in HCWs, especially with a decrease in willingness over the time, this finding emphasize the need to pay attention to the factors affecting this issue and interventions to promote HCWs’ intention to receive vaccines to reduce the spread of COVID-19 disease transmission and burden. These findings can provide useful information for policymakers to improve the use of the COVID-19 vaccine to maintain health and to prevent HCWs and the whole community from COVID-19 disease.

## Data Availability

All data generated or analysed during this study are included in this published article.

## Abbreviations

HBM: Health Belief Model
HCWs: Health Care Workers
ICC: Intraclass Correlation Coefficient
